# Ruxolitinib-corticosteroid as first-line therapy for newly diagnosed high-risk acute graft versus host disease: study protocol for a multicenter, randomized, phase II controlled trial

**DOI:** 10.1186/s13063-022-06426-2

**Published:** 2022-06-06

**Authors:** Liping Dou, Bo Peng, Xin Li, Lu Wang, Mingyu Jia, Lingmin Xu, Fei Li, Daihong Liu

**Affiliations:** 1 Department of Hematology, The Fifth Medical Center of Chinese PLA General Hospital, No. 28 Fuxing Road, Haidian District, Beijing, 100853 China; 2 Medical School of Chinese PLA, Beijing, 100853 China; 3 The Second School of Clinical Medicine, Southern Medical University, Guangzhou, 510515 China; 4 Department of Quality Control, The First Medical Center of Chinese PLA General Hospital, Beijing, 100853 China

**Keywords:** Acute graft versus host disease, Ruxolitinib, First-line therapy, Biomarker

## Abstract

**Background:**

The response rate of the first-line therapy with corticosteroid for acute graft versus host disease (aGVHD) is about 50%, and steroid-refractory disease is associated with high mortality. The improved response rate to the first-line therapy of newly diagnosed aGVHD patients would result in therapeutic benefits. Ruxolitinib, a selective Janus kinase (JAK) 1/2 inhibitor, has been approved for the treatment of steroid-refractory acute GVHD. The addition of ruxolitinib to the first-line therapy may improve the efficacy of corticosteroids.

**Methods:**

This investigator-initiated, open-label, multicenter, prospective randomized, and controlled two-arm phase II study compares the efficacy and safety of ruxolitinib combined with 1 mg/kg methylprednisolone versus 2 mg/kg methylprednisolone alone in newly diagnosed aGVHD patients. Patients with intermediate or high-risk aGVHD, as defined by the Minnesota aGVHD high-risk score and biomarker algorithm, are eligible for this study. A total of 198 patients will be randomized at a 1:1 ratio and assigned a GVHD risk (intermediate versus high risk) and disease status before transplantation (complete remission versus no complete remission). The primary endpoint is the overall response rate on day 28, which is defined as an improvement of at least one stage in the severity of aGVHD in one organ without deterioration in any other organ or disappearance of any GVHD signs from all organs without requiring new systemic immunosuppressive treatment. The secondary objectives consist of response time, response duration, overall survival, disease-free survival, non-relapse mortality, failure-free survival, and changes in serum levels of proinflammatory cytokines and GVHD-related biomarkers.

**Discussion:**

This open-label, multicenter, two-arm randomized trial will evaluate whether the addition of ruxolitinib combined with corticosteroid is superior to corticosteroid alone in newly diagnosed high-risk aGVHD.

**Trial registration:**

ClinicalTrials.gov NCT04061876 (version number: 2019.5.18). Registered on July 16, 2019

**Supplementary Information:**

The online version contains supplementary material available at 10.1186/s13063-022-06426-2.

## Background

Acute graft-versus-host disease (aGVHD) remains a major transplantation-related complication despite standard prophylaxis [1–32]. Systemic corticosteroid therapy is the first-line treatment for newly diagnosed aGVHD patients [4, 5]. However, steroid-refractory aGVHD (SR-aGVHD) occurs in approximately 35–50% of patients and is associated with high mortality [1, 6–8], indicating an intrinsic variability of glucocorticoid sensitivity at aGVHD onset [9]. The steroid resistance in recurrent aGVHD further supports this speculation [4]. Enhancing glucocorticoid sensitivity in newly diagnosed aGVHD patients for the first-line therapy could result in therapeutic benefits. Methylpredinisone at a dose of 2 mg/kg also renders patients susceptible to infections, osteoporosis, and various metabolic disturbances [8, 10]. Thus, there is an urgent requirement for novel active first-line treatments to improve efficiency and decrease the side effects in newly diagnosed aGVHD patients.

The pathogenesis of aGVHD is complicated. Early tissue injuries, the activation of innate immune cells and donor T cells, and the subsequent immune response lead to healthy tissue damage [11, 12]. Janus kinases (JAKs) are intracellular signaling molecules that regulate the activities of immune cells, including neutrophil cells, antigen-presenting cells, T cells, and B cells underlying GVHD, and therefore regulate aGVHD pathogenesis [11, 13]. Cytokines and chemokines such as interleukin (IL)-1, interferon-gamma, IL-2, tumor necrosis factor, IL-6, IL-17, and IL-33 are involved in GVHD pathogenesis [5, 11, 14]. Intracellular signaling downstream of multiple cytokines related to aGVHD is partially transduced by JAK signaling pathways [15].

The clinical stage of aGVHD is defined according to each involved organ’s clinical assessment of symptoms. The organ stages are totaled in an overall grade (I–IV) [16]. Grade III/IV aGVHD is associated with a high mortality rate (50–70%) and the possibility of steroid resistance. Serum biomarkers have also emerged as an additional potential measurement of aGVHD severity [17, 18]. The Mount Sinai Acute GVHD International Consortium (MAGIC) is a group of 25 stem cell transplantation centers conducting GVHD research. It has established an algorithm that combines two biomarkers, namely ST2 and REG3α. The MAGIC algorithm probability (MAP) predicts the response to first-line treatment of corticosteroids and 6-month non-relapse mortality (NRM). When measured at aGVHD onset, the MAP could categorize the patients into three risk groups, each group with significantly different risk of NRM. The proportion of patients resistant to treatment at week 4 was higher in the high MAP risk group than in the low-risk group (67% vs. 30%, *p* = 0.03) [14, 19]. Therefore, novel and active first-line therapies are urgently required in patients with high-risk aGVHD prone to steroid resistance [9, 20].

Ruxolitinib, a selective inhibitor of Janus kinase (JAK) 1/2, is the first Food and Drug Administration-approved medication for SR-aGVHD [4, 15, 21]. The REACH2 phase 3 randomized trial showed that in patients with SR-aGVHD, the overall complete response (CR) at day 28 was higher in the ruxolitinib group than in the control group (62% vs. 39%; *p* < 0.001). The durable overall response rate (ORR) at day 56 was significantly higher in the ruxolitinib group than in the control group (40% vs 22%; *p* < 0.001) [22]. The incidence of infection was similar in ruxolitinib and control therapy, with grade 3 severity of 22% and 19%, respectively. The incidence of cytomegalovirus (CMV) reactivation was 26%, and Epstein–Barr virus (EBV) reactivation was 6% in the ruxolitinib group [22]. Delgado et al. showed that ruxolitinib enhances cell sensitivity to dexamethasone-induced apoptosis in vitro. The combination of corticosteroid and ruxolitinib alters the balance between pro- and anti-apoptotic factors in cells with corticosteroid resistance [23]. Thus, the addition of ruxolitinib may improve the efficacy of corticosteroids in SR-aGVHD patients. Treatment with methylprednisolone at 2 mg/kg/day or prednisone at 2.0 to 2.5 mg/kg/day is the standard first-line systemic therapy for acute GVHD. Methylprednisolone is the most commonly used corticosteroid. Also, other types of steroids are available if administered at an equivalent steroid dose. In the present study, methylprednisolone was adopted to standardize the research.

We previously reported the results of the first-line treatment for newly diagnosed aGVHD patients with a combination of different doses of ruxolitinib with corticosteroid [4, 24]. This phase I dose-finding study investigated the optimal dose of ruxolitinib combined with corticosteroid (ClinicalTrials.gov Identifier: NCT04397367). The average accumulated dose of methylprednisolone was 17.6 mg/kg (standard deviation [SD] = 8.2) with a mean withdrawal time of 42.6 ± 16.8SD days. Patients were treated with three different doses of ruxolitinib: 10 mg twice daily for the first three patients, 5 mg twice daily for the following 12 patients, and 5 mg once daily for the remaining 17 patients. In the first three patients, intolerable hematologic toxicity related to 10 mg twice daily ruxolitinib was observed. CMV and EBV diseases were seen in 2 of 12 patients who received a dose of ruxolitinib 5 mg twice daily, with one suffering from CMV infection and another developing post-transplant lymphoproliferative disorder. As observed in the last 17 patients, 5 mg/day ruxolitinib combined with methylprednisolone (1 mg/kg/day) was well tolerated with decreased CMV reactivation and promising signals of efficacy as demonstrated by a 28-day ORR of 82.05% [24]. After the initiation of the novel first-line therapy, remission of aGVHD in all patients occurred at a median time of 3.2 (interquartile range [IQR], 1–7) days. Of all patients, 10 patients (31.2%) developed recurrent aGVHD after complete remission. The causes of recurrent GVHD included ruxolitinib and cyclosporine reduction (*n* = 8) and therapeutic donor lymphocyte infusion (DLI; *n* = 1). The relapse rate of primary disease in all patients with aGVHD was 15.6% (5/32). With a median follow-up of 260 days, the 1-year overall survival (OS) and disease-free survival (DFS) were 73.4% (CI 56.6–95.1%) and 61.2% (95% CI 37.4–95.6%), respectively. Cyclosporine A (CSA) is tapered over 60 days in the ruxolitinib group with a durable complete response. The short duration of CSA may accelerate immune reconstitution, which is good for reducing relapse of primary disease and infection. These data suggested that a regimen of ruxolitinib plus corticosteroids may improve the long-term outcomes of patients with newly diagnosed aGVHD with tolerance.

## Methods

### Design

We aim to further evaluate whether 5 mg/day ruxolitinib plus 1 mg/kg methylprednisolone is superior to 2 mg/kg methylprednisolone alone in aGVHD.

**Fig. 1:**
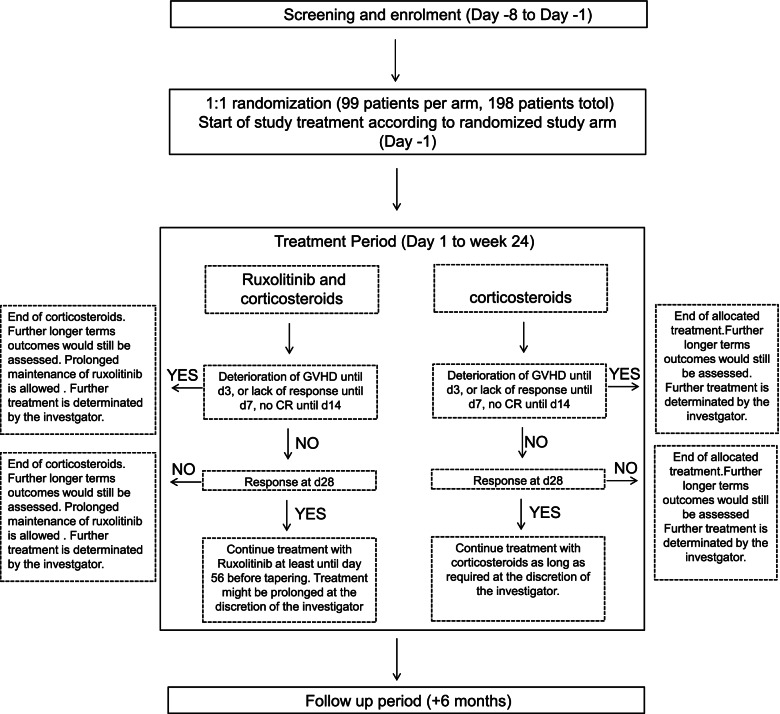
Flow chart illustrating the treatment schedule for enrolled patients

This trial is an open-label, multicenter, prospective randomized two-arm phase II study comparing the efficacy and safety of ruxolitinib plus methylprednisolone (1 mg/kg) vs. methylprednisolone (2 mg/kg) alone (Fig. 1). The planned sample size is 198 patients for internet-based randomization. The biomarkers of aGVHD, such as sST2, sTNFR1, IL-6, IL-8, and REG3α will be tested at eight time points (pre-transplantation, aGVHD onset, before treatment, and on days 7, 14, 28, 60, and 90 after treatment). Patients with newly diagnosed intermediate-risk or high-risk aGVHD, as defined by Minnesota aGVHD risk score and the biomarker score, will be included. The treatment efficacy will be evaluated with respect to the level of biomarkers and clinical manifestations. Written informed consent must be obtained from the patients before enrollment in the study. The consent form and related materials are available from the corresponding author on request.

### Study objectives and endpoints

#### Primary endpoint

##### Day 28 ORR

The day 28 ORR is defined as the proportion of patients in each treatment group achieving a partial response (PR) or CR without requiring additional immunosuppressive drugs on day 28 after enrollment [25]. PR and CR are defined as improvements of at least one stage of severity of aGVHD in one involved organ, without deterioration in any other target organ (PR) or disappearance of any GVHD signs from all target organs (CR) without requiring additional systemic immunosuppressive therapy [25]. ORR is used to assess whether the response rate to the combination therapy is better than that of steroid alone treatment by comparing the aGVHD burden at specific time points based on clinical manifestations [26].

#### Secondary endpoints

##### Durable ORR on day 56

Durable ORR on day 56 is defined as the proportion of patients in each arm whose response on day 28 lasts until day 56 after randomization.

##### Six-month duration of response (DOR)

DOR is defined as the time from first response to GVHD progression, recurrence, or death, assessed in all participants who acquire CR and are still enrolled in the study until day 180 after enrollment. Death without aGVHD recurrence or progression and relapse is considered a competing event.

##### Response duration

The response duration is defined as the time from the first response to the first recurrence/progression of aGVHD or additional immunosuppressants for GVHD. Competing events are defined as death without aGVHD recurrence or occurrence of chronic GVHD (cGVHD), and if neither occurs, the observation is treated as censored data. However, the inflammation may still exist after GVHD signs disappear. Signs of aGVHD may reoccur after a certain period of time, even its previous signs disappeared completely. Duration of response for aGVHD after treatment is required to evaluate the durability of response [16].

##### NRM

NRM is defined as the time from enrollment to death due to any causes other than hematologic disease relapse. NRM and OS can be used to compare the survival benefits of the two treatments for aGVHD.

##### Cumulative incidence of relapse

Relapse is defined as the reappearance of a primary hematologic disease after transplantation. The cumulative incidence of relapse is estimated in the competing risk framework and death without relapse as the competing event.

##### DFS

DFS is defined as the time from enrollment to relapse of primary disease or death from any causes, whichever occurred first. OS is defined as the time from randomization to death due to any causes. DFS and relapse rate are employed to assess the impact of two treatments on disease relapse. Retrospective data demonstrated the inverse correlation between the GVHD and the relapse risk after transplantation.

##### Failure-free survival

Failure-free survival (FFS) refers to the time from randomization to disease relapse or progression, non-relapse mortality, or the addition of new therapy for aGVHD.

##### Rate of recurrent aGVHD

The rate of recurrent aGVHD is defined as the proportion of patients in each arm whose aGVHD recurs after complete remission. Recurrent aGVHD is defined as the reappearance of the clinical symptoms of aGVHD after complete remission of previous aGVHD manifestations that reflect the response to treatment.

##### Safety

All adverse effects that occur in the study period should be recorded.

### Target population

Eligible patients with aGVHD graded according to the modified Glucksberg criteria and untreated with systemic aGVHD therapy will be enrolled in this study. The grade of aGVHD response to treatment will be determined by a special team of three experienced doctors from the transplantation centers. All centers are experienced in the management of GVHD and stem cell transplantation. However, some conditions, such as poor patient compliance to doctor’s orders, will interfere with patients’ participation in the study. Such patients should not be included in the study at the judgment of the research team. Throughout the study period, the directors of the centers would supervise the implementation and review of the research documents. Patients who fulfill all inclusion criteria could be included in the study.

### Inclusion criteria

First, 14–65-year-old candidates for their first allogeneic hematopoietic stem cell transplantation using bone marrow and/or peripheral blood for hematological diseases. Second, eligible patients must develop a de novo aGVHD, defined as intermediate or high risk using the Minnesota aGVHD risk score and biomarker algorithm.

### Exclusion criteria

Patients who fulfill one of the following exclusion criteria are not eligible for the present study: (1) patient-related criteria: female patients with pregnant or breast-feeding; acquired immune deficiency syndrome (AIDS); hepatitis B/C infection; uncontrolled bacterial, fungal, or viral infections; allergies to ruxolitinib; or vital organ dysfunction unrelated to GVHD, including persistent bilirubin abnormalities or severe heart and respiratory diseases; (2) transplant-related criteria: relapse of the primary disease or graft rejection after transplantation; (3) GVHD-related criteria: presence of cGVHD or associated clinical signs or DLI-induced aGVHD or interferon; (4) drug-related criteria: previous history of JAK1/2 inhibitor treatment after transplantation, except JAK inhibitors administered before the transplantation; and (5) patients with poor compliance should not be included in the study based on the judgment of the research team, for example, if they refused to take medicine at will many times during the previous treatment.

### Randomization

The eligibility of patients is determined by study site staff. Then, the participants who meet the enrollment criteria will be randomized to receive one of the two treatments in a 1:1 ratio and stratified by disease status before transplantation (complete remission vs. non- complete-remission) and risk of aGVHD (intermediate risk vs. high-risk). Stratified permuted block randomization lists will be used to generate the stratified randomization. Randomization is implemented through an interactive web-based response system independent of study site staff and investigators. The codes are generated independently of the study by a statistician. The next assignment in the random sequence remained concealed because the treatment was assigned remotely. Treatment allocations are not masked to investigators or participants as CSA and methylprednisolone dosage are different between the two groups. The study staff who conducted the data analysis and assessments of outcomes will be masked for treatment allocations.

### Recruitment procedures

The participants will be recruited in one of the transplantation centers located in Beijing, Dalian, Changchun, Shenzhen, Shijiazhuang, and Jinan. All these centers are highly experienced in the management of GVHD and stem cell transplantation. Throughout the study period, the directors of the centers supervise the implementation and review of the research documents. An independent research assistant recruits the eligible participants. In each transplant center, the doctors participating in this study are trained uniformly for treatment, taking informed consent, and study visits. The trained nurses coordinate the study visits and record the data. Each center will recruit patients (enrollment from August 2019 to January 2023, five patients from all centers every month). No financial incentives were provided to trial investigators or participants for enrollment.

The transplantation knowledge was provided to the patients and their relatives in order to emphasize the management of transplantation and promote participant retention. Also, a standardized follow-up booklet is granted to each enrolled patient for recording and reminding the detailed follow-up items and time points. An in-time communication between investigators and subjects by mobile phones will facilitate the completion of follow-up.

### Study treatment

#### Ruxolitinib plus corticosteroids treatment (ruxolitinib group)

The initial dose of methylprednisolone is 1 mg/kg/day intravenously given in this arm for at least 7 days. The dosage of ruxolitinib is 5 mg once daily orally. CSA will be administered intravenously at a dose of 2 mg/kg twice daily, targeting minimum concentration levels of 150–250 ng/mL. If GVHD response to treatment is assessed as PR/CR at 7 days, methylprednisolone will be tapered, followed by reduction and discontinuation of CSA and then ruxolitinib. The reduction of the methylprednisolone in the ruxolitinib group is gradual, suggesting it be tapered off over 6 weeks as follows: dosage reduced to 0.6 mg/kg/day, 0.4 mg/kg/day, 0.3 mg/kg/day, 0.25 mg/kg/day, and 0.18 mg/kg every 5 days, followed by 0.1 mg/kg/day at week 4, 0.1 mg/kg every other day after 5 days, and stopped at week 6. After steroid discontinuation and CR or stable PR maintained for 6 weeks, CSA is tapered over 60 days. After CSA discontinuation and no presence of recurrent GVHD, ruxolitinib is tapered off over 90 days, which is totally maintained for approximately 6 months [2].

In view of the durable response and safety observed in previous ruxolitinib studies, the clinical benefits of ruxolitinib outweigh the risks of treatment. Retrospective data indicated that prolonged maintenance of ruxolitinib is allowed even after refractory GVHD, and responses in aGVHD can be observed at 11 weeks after ruxolitinib treatment [4, 15]. Prolonged maintenance of ruxolitinib is allowed in the case of recurrent aGVHD or refractory aGVHD (the progression of GVHD after 3 days of therapy, no improvement within 7 days, or no CR after 14 days of therapy) after ruxolitinib plus corticosteroid treatment [4]. If patients in the ruxolitinib group do not reach the CR by day 28, ruxolitinib will also be continued beyond 6 months in the event of no contraindications. Tapering of ruxolitinib is permitted after day 56 in patients who have a response to the second-line therapy for recurrent aGVHD or refractory aGVHD. An additional follow-up through at least day 180 is required to determine the durability of treatment responses [27]. During the trial, drug interactions should be examined first before adding new agents in order to reduce the potential effects of other drugs on ruxolitinib. Fluconazole > 200 mg/day is prohibited as it disrupts the metabolism of ruxolitinib.

#### Corticosteroid treatment (corticosteroids group)

The initial dose of methylprednisolone is 2 mg/kg/day given twice daily for at least 7 days and then reduced [8]. The dosage of methylprednisolone in the corticosteroids group is decreased gradually after CR and tapered off over 10 weeks as follows: dosage reduced from 1 mg/kg/day to 0.6 mg/kg/day, 0.4 mg/kg/day, 0.3 mg/kg/day, 0.25 mg/kg/day, and 0.18 mg/kg every 7 days and then 0.1 mg/kg/day at week 4, 0.1 mg/kg every other day after 5 days, and stopped at week 10. CSA was administered intravenously at a dose of 2 mg/kg twice daily, targeting minimum concentration levels of 150–250 ng/mL. The recommended duration of CSA is 6 months.

In both arms, for refractory aGVHD, i.e., the progression of GVHD after 3 days of therapy, no improvement within 7 days, or no CR after 14 days of therapy, the second-line therapy will be started. For refractory aGVHD or recurrent aGVHD, basiliximab may be used as second-line therapy, and other alternative drugs including methotrexate or mesenchymal stem cells may be used at the researcher’s decision. For refractory aGVHD, methylprednisolone is discontinued and CSA is continued in both arms. Prolonged maintenance of ruxolitinib is used in the ruxolitinib group. A standardized follow-up booklet is given to each enrolled patient for recording the dose, frequency, and administration method of the drugs.

### Adverse events (AEs)

Clinicians assess the AEs according to the Common Terminology Criteria for Adverse Events (CTCAE) v.4.0. Cytopenias are identified based on CTCAE grades. Anemia: grade 1, hemoglobin (Hgb) < lower limit of normal (LLN)–10.0 g/dL; grade 2, Hgb < 10.0–8.0 g/dL; grade 3, Hgb < 8.0 g/dL, transfusion indicated; and grade 4, life-threatening consequences, urgent intervention indicated. Neutropenia count decreased: grade 1, < LLN–1500/mm^3^; grade 2, < 1500–1000/mm^3^; grade 3, < 1000–500/mm^3^; and grade 4, < 500/mm^3^. Platelet count decreased: grade 1, < LLN–75,000/mm^3^; grade 2; < 75,000–50,000/mm^3^; grade 3, < 50,000–25,000/mm^3^; and grade 4, < 25,000/mm^3^. The patient’s tolerance to the drug is closely monitored, and any AEs would be recorded. During the trial, if the participant has any serious AEs caused by the study treatment, the researcher will promptly report to the principal investigator and the relevant department of the hospital. Appropriate compensation will be provided to the subjects injured due to participation in the trial.

### GVHD biomarker Luminex assays

Peripheral blood samples are collected at predetermined peri-transplant time points (pre-transplantation, aGVHD onset, before treatment, and on days 7, 14, 28, 60, and 90 after enrollment) [17, 28, 29]. Designated personnel are responsible for collecting and transporting these blood samples that are shipped to Bofurui Biolab for MAP analyses for ST2 and REG3a by flow cytometry. Luminex Assay Human Premixed Multi-Analyte Kits (R&D, MN, USA, Catalog No. LXSAHM-05) are used for the measurement of ST2 and REG3a, according to the manufacturer’s protocol. Samples (diluted as 1:2) and standards are run in duplicate, the absorbance is measured (Luminex 200), the data are estimated using versionXponent_4.2 (Luminex 200), and MAP is calculated accordingly.

### Assessments and data collection

Assessments will be performed at three stages: (1) prior-treatment screening to assess the inclusion and exclusion criteria (once from days − 7 to − 1 before medication); (2) treatment evaluation to determine the response and adjustment of medication maintenance (twice weekly for week 1, once weekly from weeks 2 to 6, once every 2 weeks from weeks 7 to 12, and once every month from weeks 13 to 24 after the start of medication n); (3) post-treatment evaluation for follow-up (every 2 months for 6 months). Routine assessments for each evaluation included physical check-ups (contributing to ORR, durable ORR on day 56, 6-month duration of response, the response duration, DFS, FFS, and the recurrence rate of aGVHD), standard laboratory tests (contributing to relapse, NRM, DFS, FFS, and the recurrence rate of aGVHD), GVHD grading (contributing to ORR, durable ORR on day 56, 6-month duration of response, the response duration, FFS, and the recurrence rate of aGVHD), EBV and CMV-PCR (contributing to adverse events), and GVHD medication (contributing to ORR, durable ORR on day 56, 6-month duration of response, and the recurrence rate of aGVHD). GVHD biomarkers are detected during the treatment phase at exact time points (pre-transplantation and aGVHD onset) and are trial-specific. Additionally, the AEs are assessed during every visit after the start of the medication. The data can be found in Supplementary Table 1 and Fig. 1. Accurate and reproducible reports for the diagnosis and grade of acute GVHD are critical for the evaluation of therapies and biomarkers in GVHD study.

### Data management

The participant’s baseline data, trial data, and follow-up data will be recorded together in the case report form by the operator and evaluator. The patients are identified by their number to remain anonymous. For participants who discontinued the study, the specific reason and time of withdrawal will be recorded in addition to other regularly monitored indicators. The designated inspector will check each item in the database and verify the inconsistent values of the original questionnaire. The investigators will not be able to access the dataset, and no interim analysis is carried out for this experiment. All trial-related data will be maintained at the Department of Hematology, Chinese PLA General Hospital. The personal information and privacy of all participants would be confidential.

### Statistical considerations

The sample size is calculated according to the primary endpoint (ORR) of the study. Based on our published phase I study of 32 aGVHD patients who received steroids as first-line therapy, an expected proportion of 55% for the patients treated with corticosteroids only was established [30]. Based on our published data of patients with aGVHD grades I–IV who received steroid-ruxolitinib (5 mg/day) as first-line therapy, the ORR was 82.05% [24]. Patients with aGVHD grade I were also included in the phase I analysis. Thus, an expected proportion of 75% for the patients treated with steroid-ruxolitinib (5mg/day) was established. This study is planned to detect a response difference between treatment arms at a two-sided significance level *α* = 5% with a power of 1−*β* = 80%. The sample size was estimated using the PASS software based on the primary endpoint (ORR). The calculated sample size is shown in Fig. 1 (N1 = 99, N2 = 99); allowing a withdrawal rate of 10%, 198 patients (99/group) will be required.

The baseline characteristics of patients will be reported in detail. The discrete variables are described by a median with IQR. The mean and standard deviation (SD) is used for quantitative variables. A logistic regression model is established as the primary study endpoint of GVHD treatment, as described above. In this model, the treatment grouping factor (ruxolitinib plus corticosteroids vs. corticosteroids alone), stratified variables of aGVHD risk (high-risk vs. intermediate risk), and disease status before transplantation (complete remission vs. non-complete remission) are included as covariates. The subgroup analysis is based on the significant effect of biomarker risk stratification on survival outcomes.

The estimated rates with two-sided 95% confidence intervals (CIs) are used for secondary endpoints (proportion of CR patients and patients who stopped treatment). The ORR on day 28 is calculated with its 95% CI. The Kaplan–Meier method is used to estimate the DOR, OS, and DFS, and the log-rank test is used to evaluate the difference between the groups [21]. The cumulative incidence of NRM and relapse is estimated using a competing risk model and compared using the Fine and Gray test. The Cox proportional hazard regression model is used for multivariable regression analysis for OS and DFS. The multivariable regression analysis for NRM is performed using the Fine–Gray proportional hazard regression for competing events. Potential risk factors considered in the regression analysis include primary disease, cytogenetic risk, age and gender of the donor and recipient, graft source, treatment arm, aGVHD risk, and disease status before transplantation. The cumulative incidence of recurrent aGVHD is analyzed using the Fine and Gray test in a competing risk framework. The threshold for statistical significance is set at 0.05, and all tests are two-sided. All analyses are carried out using the SPSS 22.0 software (IBM Corporation, Armonk, NY, USA) and R version 4.1.2 (www.cran.r-project.org). The treatment-induced differences in OS and DFS will be based on the Cox proportional hazard regression model. The reported treatment difference in NRM and relapse will be based on the multivariate analysis using Fine–Gray proportional hazard regression.

### Handling of missing data

All randomized participants will be included in the primary analysis of all outcomes. The proportion of missing values on the primary and secondary outcomes would be < 10%. Thus, a secondary analysis should be considered using multiple imputations and present best-case/worst-case scenarios if ignoring the missing data is not plausible.

#### Standard protocol items

##### Recommendations for interventional trials (SPIRIT)

This protocol has been designed according to the SPIRIT guidelines. The SPIRIT checklist is provided in Additional file 1.

## Discussion

This randomized trial aimed to determine whether the addition of ruxolitinib to corticosteroid as the first-line therapy for newly diagnosed aGVHD is associated with an increased complete response rate on day 28, thereby improving the transplant outcomes. If this is confirmed, a reexamination may be required for the current first-line treatment strategies in the newly diagnosed aGVHD to improve the CR rate.

It has been suggested that clinically meaningful and ongoing responses can be induced by the inhibition of JAK1/2 with ruxolitinib in patients with newly diagnosed SR-GVHD [7, 15, 21, 22]. To address the benefits of ruxolitinib plus corticosteroid vs. corticosteroid alone treatments, a randomized trial is required. This study is the first prospective multicenter clinical trial focused on comparing the efficacy of ruxolitinib plus corticosteroid vs. corticosteroid alone for the treatment of newly diagnosed aGVHD [25]. In both arms, the second-line therapy will be started for refractory aGVHD. Retrospective data indicated that responses in aGVHD can be observed at 11 weeks after ruxolitinib treatment [4, 15]. Thus, patients in the ruxolitinib group not meeting the primary endpoint at day 28 will maintain ruxolitinib therapy along with second-line therapy [33]. Thus, ruxolitinib can be used as long as the patient benefits from the treatment. The dose of ruxolitinib can be modified because of hematologic adverse events, drug-related, or non-hematologic adverse events unrelated to GVHD [21, 34].

This open-label, multicenter, two-arm randomized trial evaluates whether the addition of ruxolitinib plus corticosteroids is superior to corticosteroids alone in the newly diagnosed high-risk aGVHD.

## Trial status

This study will be performed with the principles of the Declaration of Helsinki (Fortaleza, Brazil, October 2013) and current Chinese legislation on clinical trials. This study will be conducted according to International Conference on Harmonization/Good Clinical Practice Standards. This study has been approved by the Clinical Research Ethics Committee of Chinese PLA General Hospital, Beijing, China, and it has been registered on ClinicalTrials.gov (Identifier: NCT04061876). The outputs from this study include conference presentations, community reporting, and journal publications and do not identify participants. The protocol version number is S2019-177-01. The recruitment began on August 25, 2019, to January 1, 2023. The anticipated end date is July 1, 2023.

## Supplementary Information

**Additional file 1.** Standard Protocol Items: Recommendations for Interventional Trials (SPIRIT) 2013 checklist.

**Additional file 2: Supplementary Table 1.** Study procedure and Flow Chart (treatment phase prior to end of study).

## Data Availability

The datasets generated and analyzed during the current study are available from the corresponding author on reasonable request.

## Abbreviations

aGVHD: Acute graft versus host disease
AE: Adverse event
CI: Confidence interval
CSA: Cyclosporine A
cGVHD: Chronic acute graft versus host disease
CR: Complete remission
CMV: Cytomegalovirus
CYP: Cytochrome P450
DFS: Disease-free survival
DLI: Donor lymphocyte infusion
DMC: Data monitoring committee
DAMPs: Damage-associated molecular patterns
EBV: Epstein–Barr virus
FFS: Failure-free survival
GVHD: Graft versus host disease
Hgb: Hemoglobin
IL: Interleukin
IQR: Interquartile range
JAK: Janus kinase
LLN: Lower limit of normal
MAGIC: Mount Sinai Acute GVHD International Consortium
MAP: MAGIC algorithm probability
NRM: Non-relapse mortality
OS: Overall survival
ORR: Overall response rate
PR: Partial response
PCR: Polymerase chain reaction
PAMPs: Pathogen-associated molecular patterns
SR-aGVHD: Steroid-refractory acute graft versus host disease
sST2: Soluble suppression of tumorigenesis-2
sTNFR1: Soluble tumor necrosis factor receptor 1
SD: Standard deviation
REG3α: Regenerating islet-derived 3-alpha
TNF: Tumor necrosis factor
