# Supplementary material for: Ruxolitinib-corticosteroid as first-line therapy for newly diagnosed high-risk acute graft versus host disease: study protocol for a multicenter, randomized, phase II controlled trial
Source: Trials. 2022 Jun 6;23:470. doi: 10.1186/s13063-022-06426-2 (PMC9169300; doi:10.1186/s13063-022-06426-2)
Supplement: Supplementary file 2 — Additional file 2: Supplementary Table 1. Study procedure and Flow Chart (treatment phase prior to end of study). [file 13063_2022_6426_MOESM2_ESM.docx]

Supplementary Table 1: Study procedure and Flow Chart (treatment phase prior to end of study)

| **Assessments** | **Screening**  **Examination**  -8 days to day -1;  **Randomisation** day -1 | **Biweekly treatment period,**  week 1  *(± 2 days)* | **Weekly**  **treatment**  **period**  from week 2 to  week 6 *(± 2 days)* | | | | | | | | **Biweekly**  **treatment**  **period**  from week 7 to  week 12 *(± 2 days)* | | | **Monthly**  **treatment period**  from week 13 to  week 24  *(± 2 days)* | | | **Additional**  **treatment**  **phase^[[1]](#endnote-1)^**  every two  months  *(± 14 days)* | **End of**  **Study** |
| --- | --- | --- | --- | --- | --- | --- | --- | --- | --- | --- | --- | --- | --- | --- | --- | --- | --- | --- |
|  |  | Week 1,  day 3,7 | Week 2,  day 8 | | Week 3,  day 15 | | Week 4,  day 22 | | Week 5,  day 29 | Week 6,  day 36 | Week 8,  day 50 | Week 10,  day 64 | Week 12,  day 78 | Week 16,  day 106 | Week 20,  day 134 | Week 24,  day 162 |  | Month 6 |
| Informed Consent | x |  |  | |  | |  | |  |  |  |  |  |  |  |  |  |  |
| Patient eligibility | x |  |  | |  | |  | |  |  |  |  |  |  |  |  |  |  |
| Disease status before transplantation^[[2]](#endnote-2)^ | x |  |  | |  | |  | |  |  |  |  |  |  |  |  |  |  |
| Randomisation | x |  |  | |  | |  | |  |  |  |  |  |  |  |  |  |  |
| Serum sample ST2 and REG3α (central)^[[3]](#endnote-3)^ | x | x | x | | x | | x | |  | x |  | x | x |  |  |  |  |  |
| **Ruxolitinib/Steroids or Steroids only** |  | ***continues every day from day 1*** | | | | | | | | | | | | | | | x |  |
| Physical examination | x | x | | x | | x | | x | x | x | x | x | x | x | x | x | x | x |
| Weight | x | x | | x | | x | | x | x | x | x | x | x | x | x | x | x | x |
| Clinical GVHD grading^[[4]](#endnote-4)^ | x | x | | x | | x | | x | x | x | x | x | x | x | x | x | x | x |
| Gut biopsy (optional)^[[5]](#endnote-5)^ | x |  | |  | |  | |  |  |  |  |  |  |  |  |  |  |  |
| Skin biopsy (optional)^[[6]](#endnote-6)^ | x |  | |  | |  | |  |  |  |  |  |  |  |  |  |  |  |
| Laboratory(Haematology, clinical chemistry)^[[7]](#endnote-7)^ | x | x | | x | | x | | x | x | x | x | x | x | x | x | x | x | x |
| CMV PCR^[[8]](#endnote-8)^ | x | x | | x | | x | | x | x | x | x | x | x | x | x | x | x | x |
| EBV PCR^[[9]](#endnote-9)^ | x | x | | x | | x | | x | x | x | x | x | x | x | x | x | x | x |
| Serum biomarker IL-6, IL-8, TNFR1 (central)^[[10]](#endnote-10)^ | x | x | | x | | x | | x |  |  | x |  | x |  |  |  |  |  |
| Concomitant Medication^[[11]](#endnote-11)^ | x | x | | x | | x | | x | x | x | x | x | x | x | x | x | x | x |
| Adverse Events^[[12]](#endnote-12)^ |  | x | | x | | x | | x | x | x | x | x | x | x | x | x | x | x |
| Monitoring of in-hospital days^[[13]](#endnote-13)^ |  | x | | x | | x | | x | x | x | x | x | x | x | x | x | x | x |

##

1. Study treatment will be administered for 6 months or as long as the patient experiences benefit from treatment with Ruxolitinib. In this case treatment might be prolonged at the discretion of the investigator. In case of reappearance of GVHD signs after Ruxolitinib has been discontinued, treatment with Ruxolitinib may be reinstituted. At end of treatment the same assessments will be done as month 6. [↑](#endnote-ref-1)
2. Disease status before transplantation: CMV Status donor/recipient, previous treatment of the underlying disease including allogeneic transplantation, GvHD prophylaxis, corticosteroid treatment with duration, status of the underlying disease before transplantation, and earlier infectious complications during induction/consolidation. [↑](#endnote-ref-2)
3. Serum sample for ST2 and REG3α will be shipped to Bofurui Lab for central assessment (see laboratory manual). [↑](#endnote-ref-3)
4. According to Modified Glucksberg Criteria. [↑](#endnote-ref-4)
5. Gut biopsy is not mandatory and can be performed at any time before screening. Thus, time frame for biopsy is NOT limited to day -8 to day -1. [↑](#endnote-ref-5)
6. Skin biopsy is not mandatory and can be be performed at any time before screening. Time frame for biopsy is NOT limited to day -8 to day -1. [↑](#endnote-ref-6)
7. Local lab: hematology and clinical chemistry will be performed according to clinical routine. In the CRF only the following parameters will be documented: hemoglobin, leukocyte, platelets and neutrophil count. Laboratory data have to be checked by the investigator and in case of a clinically relevant abnormality an AE has to be recorded on the AE-page in the CRF. [↑](#endnote-ref-7)
8. CMV PCR to be performed once weekly from week 1 to 6, and biweekly from week 8 to 12 and monthly from week 16 to 24. In patients who never reactivated CMV then once every 2 months. In patients with CMV reactivation, it is recommended to determine the CMV copy number twice per week. For CMV monitoring and recommended preemptive treatment see assessments and data collection part of the protocol. [↑](#endnote-ref-8)
9. EBV PCR to be performed once weekly from week 1 to 6, and once every other week from week 8 to 12 and monthly from week 16 to 24. In patients who never reactivated CMV then once every 2 months. In patients with CMV reactivation, it is recommended to determine the CMV copy number twice per week. For CMV monitoring and recommended preemptive treatment see assessments and data collection part of the protocol. [↑](#endnote-ref-9)
10. The following serum biomarkers will be measured locally at the center: IL8, IL-6, TNFR1. [↑](#endnote-ref-10)
11. As Ruxolitinib is a substrate of CYP 3A4, CYP 2C9 and P-Glycoprotein, please check possible drug interactions before prescribing/administration of any new medication. **Administration of fluconazole at daily doses higher than 200 mg is prohibited.** All concomitant tumor-specific or other disease-modifying therapy including “targeted” therapies (other than investigational products) or systemic GVHD treatment administered at any time during the period starting with the signature of the Informed Consent Form (ICF) and ending with the study end, has to be thoroughly documented in the CRFs. [↑](#endnote-ref-11)
12. The AE reporting and documentation period begins with randomization and ends at the end of month 6 (for both treatment arms) or 30 days after last intake of ruxolitinib (whichever occurs later). For patients in the corticosteroids alone arm who crossed over to Ruxolitinib the reporting and documentation period ends 30 days after last intake of ruxolitinib. [↑](#endnote-ref-12)
13. Inpatient or outpatient status and duration of current and last inpatient hospitalization will be documented in the CRF. [↑](#endnote-ref-13)
